# MiR-367 regulates cell proliferation and metastasis by targeting metastasis-associated protein 3 (MTA3) in clear-cell renal cell carcinoma

**DOI:** 10.18632/oncotarget.18647

**Published:** 2017-06-27

**Authors:** Dexin Ding, Yue Zhang, Lin Wen, Jiangbo Fu, Xue Bai, Yuhua Fan, Yuan Lin, Hongshuang Dai, Qiang Li, Yong Zhang, Ruihua An

**Affiliations:** ^1^ Department of Urology, The First Affiliated Hospital of The Harbin Medical University, Harbin 150001, China; ^2^ Department of Urology, The Affiliated Tumor Hospital of The Harbin Medical University, Harbin 150001, China; ^3^ Department of Pharmacology, State-Province Key Laboratories of Biomedicine-Pharmaceutics of China, Key Laboratory of Cardiovascular Research, Ministry of Education, College of Pharmacy, Harbin Medical University, Harbin 150081, China; ^4^ Department of Biotechnology and Pharmaceutics, College of Pharmacy, Harbin Medical University-Daqing, Daqing 163319, China

**Keywords:** miR-367, MTA3, ccRCC, proliferation, metastasis

## Abstract

Clear-cell renal cell carcinoma (ccRCC) is an aggressive and malignant kidney cancer which has the worst prognosis. Although microRNAs (miRNAs) have recently been identified as a novel class of regulators in oncogenesis and metastasis, there are few studies on their participation in ccRCC. In the present study, we observed that miR-367 expression was increased in both human ccRCC tissues and cell lines. Cell proliferation was evaluated by MTT assay and 5-Ethynyl-2′-deoxyuridine (EdU) assay kit, which indicated that inhibition of miR-367 could suppress the ccRCC proliferation. Forced expression of miR-367 substantially induced cell migration and invasion evidenced by wound-healing and transwell assays, and this carcinogenesis could be abolished by miR-367 inhibitor treatment. Further analysis identified Metastasis-Associated Protein 3 (MTA3) as a direct target of miR-367. QRT-PCR and western blot results indicated the correlative expression of miR-367 and MTA3 in ccRCC tissue samples. Overexpression of MTA3 reversed miR-367-induced cell proliferation, migration and invasion. Our data uncovered a novel molecular interaction between miR-367 and MTA3, indicating a therapeutic strategy of miR-367 for ccRCC.

## INTRODUCTION

Renal cell carcinoma has the third highest mortality among all genitourinary cancers worldwide, of which clear-cell renal cell carcinoma (ccRCC) accounts for approximately 70% [1]. In 2013, approximately 65,150 new cases and 13,680 deaths were predicted to be associated with renal cell carcinoma in the United States [2]. Although early-stage ccRCC is curable by surgery resection, the prognosis for metastatic ccRCC patients is still poor. Researches have indicated that the 5-year survival rate of ccRCC patients remains no more than 10% [3]. Therefore, there is greater impetus to identify specific genes associated with tumor progression and migration so that novel therapeutic targets can optimize present prognostic systems.

MicroRNAs (miRNAs) are endogenous, small non-coding RNAs of 19–22 nucleotides in length that modulate fundamental cell processes at the post-transcriptional level [4]. Accumulating evidence has implicated miRNAs as essential regulators of ccRCC by targeting transcription factors or pivotal signal pathways [5–7]. MiR-335 can dictate ccRCC cells fate by inhibiting the proliferation and invasion through the repression of Bcl-w [8]. Nishikawa et al. clarify that tumor-suppressive miRNA-29s directly target LOXL2 inhibiting renal cell carcinoma progression [9]. In addition, researchers manifest that miR-367 elevation leads to poor prognosis in non-small cell lung cancer [10–11], accelerates breast cancer progression by binding with the 3′UTR of the calcium channel ryanodine receptor gene 3 (RYR3) [12] and promotes proliferation of hepatocellular carcinoma cell [13]. However, researches are lack in the potential role of miR-367 in ccRCC.

Metastasis-associated protein 3 (MTA3) is a kind of metastasis-associated protein, all of which serve as subunits of the Mi-2/NuRD nucleosome remodeling and deacetylase (NuRD) protein complex [14–16]. It is found that MTA3 expression level is significantly increased in hepatocellular carcinoma and identified to correlate with tumor progression and the poor prognosis [17]. Researchers also manifest that MTA3 can regulate the proteins of BAX, Cleved-Caspase-3, p-PARP and Bcl-2 to modify the progression of cellular apoptosis in NSCLCs [18]. Moreover, MTA3 is reported to be an independent and unfavorable prognostic marker [19]. However, till now, few studies focus on the expression of MTA3 and its potential association with microRNAs in ccRCC. In this study, we first describe potential roles of miR-367 in the proliferation and metastasis of ccRCC and then center on the underlying molecular mechanisms of which MTA3, one of the direct targets of miR-367, may contribute to ccRCC progression.

## RESULTS

### MiR-367 expression is elevated both *in vivo* and *in vitro*

**Figure 1:**
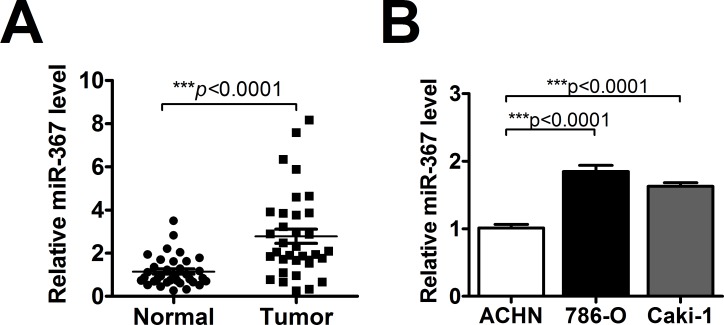
MiR-367 was highly expressed in ccRCC tissues and cells **(A)** QRT-PCR analysis of miR-367 in 35 pairs ccRCC tissues. Data are expressed as mean ± SEM. ***p<0.0001 *vs*. normal. **(B)** The expression of miR-367 was measured by qRT-PCR in human renal cell adenocarcinoma ACHN and two ccRCC cell lines (786-O and Caki-1). Data are expressed as mean ± SEM. ***p<0.0001 *vs*. ACHN.

To explore the potential role of miR-367 in ccRCC carcinogenesis, we first measured the expression of miR-367 in 35 pairs of ccRCC tissues and their matched normal renal tissues using qRT-PCR. The result showed that miR-367 expression was increased in ccRCC tissues (Figure 1A). In addition, miR-367 was also dramatically upregulated both in 786-O and Caki-1 cell lines (Figure 1B). The above results suggested that miR-367 might be involved in the progression of ccRCC.

### MiR-367 contributes to cell proliferation in ccRCC

**Figure 2:**
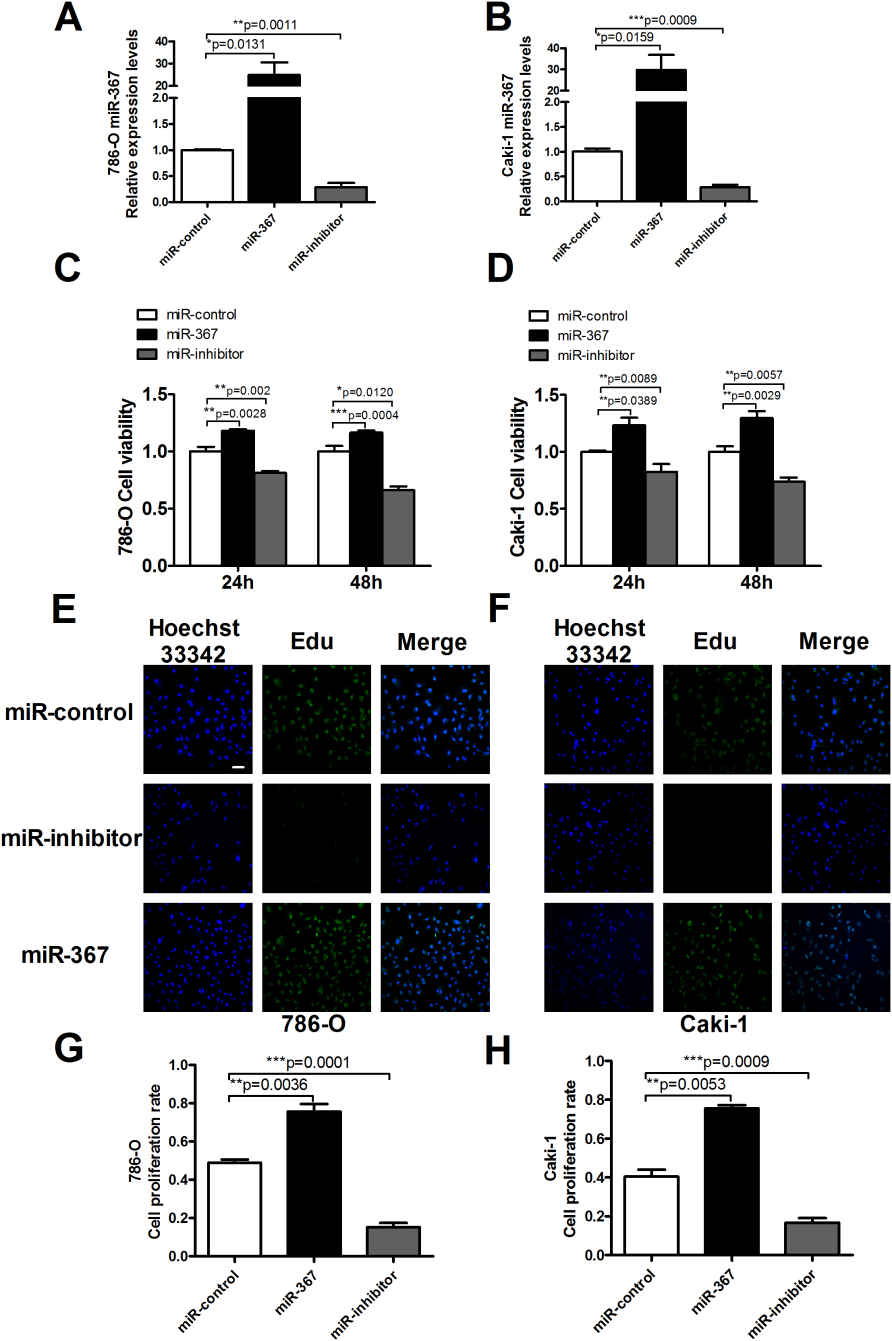
MiR-367 regulated cell proliferation in ccRCC cells **(A** and **B)** MiR-367 level was measured by qRT-PCR after transfection with miR-control (negative control), miR-367 mimics (miR-367) or miR-inhibitor in 786-O and Caki-1 cells. **(C** and **D)** Caki-1 and 786-O viabilities were detected using MTT assay. ccRCC cells were transfected with miR-367 or miR-367 inhibitor for 24 h and 48 h. **(E** and **F)** Representative images of Edu staining showing proliferation cells (stained in green). Nuclei that double labeled with EdU (green) and Hoechst 33342 (blue) were considered to be new proliferative cells. Scale bar indicates 100 μm. **(G** and **H)** Quantitative analysis of double labeled nuclei was performed. The number of double labeled nuclei was significantly increased in miR-367-treated group compared with miR-control-treated group. Data are expressed as mean ± SEM. **p<0.01 or *p<0.05 *vs*. miR-control.

To identify the function of miR-367 in ccRCC cell proliferation, miR-367 mimics (miR-367) or inhibitor were employed both in Caki-1 and 786-O cell lines. The transfection efficiency was evaluated by qRT-PCR (Figure 2A and 2B). Our analysis indicated that miR-367 overexpression dominantly increased cell viability compared with its corresponding miR-control at 24 h and 48 h time points, whereas miR-367 inhibitor markedly decreased cell viability (Figure 2C and 2D). 5-Ethynyl-2′ -deoxyuridine (EdU) assay was performed to further explore the functional role of miR-367 in ccRCC cell proliferation. The results demonstrated that more proliferative cells double labeled with EdU and Hoechst 33342 were observed in the group treated with miR-367 mimics versus miR-control, whereas a markedly reduction was seen after transfection of miR-367 inhibitor (Figure 2G and 2H). Representative images were shown in Figure 2E and 2F. These data together suggested that miR-367 could facilitate ccRCC cell proliferation.

### MiR-367 promotes ccRCC cell migration and invasion

**Figure 3:**
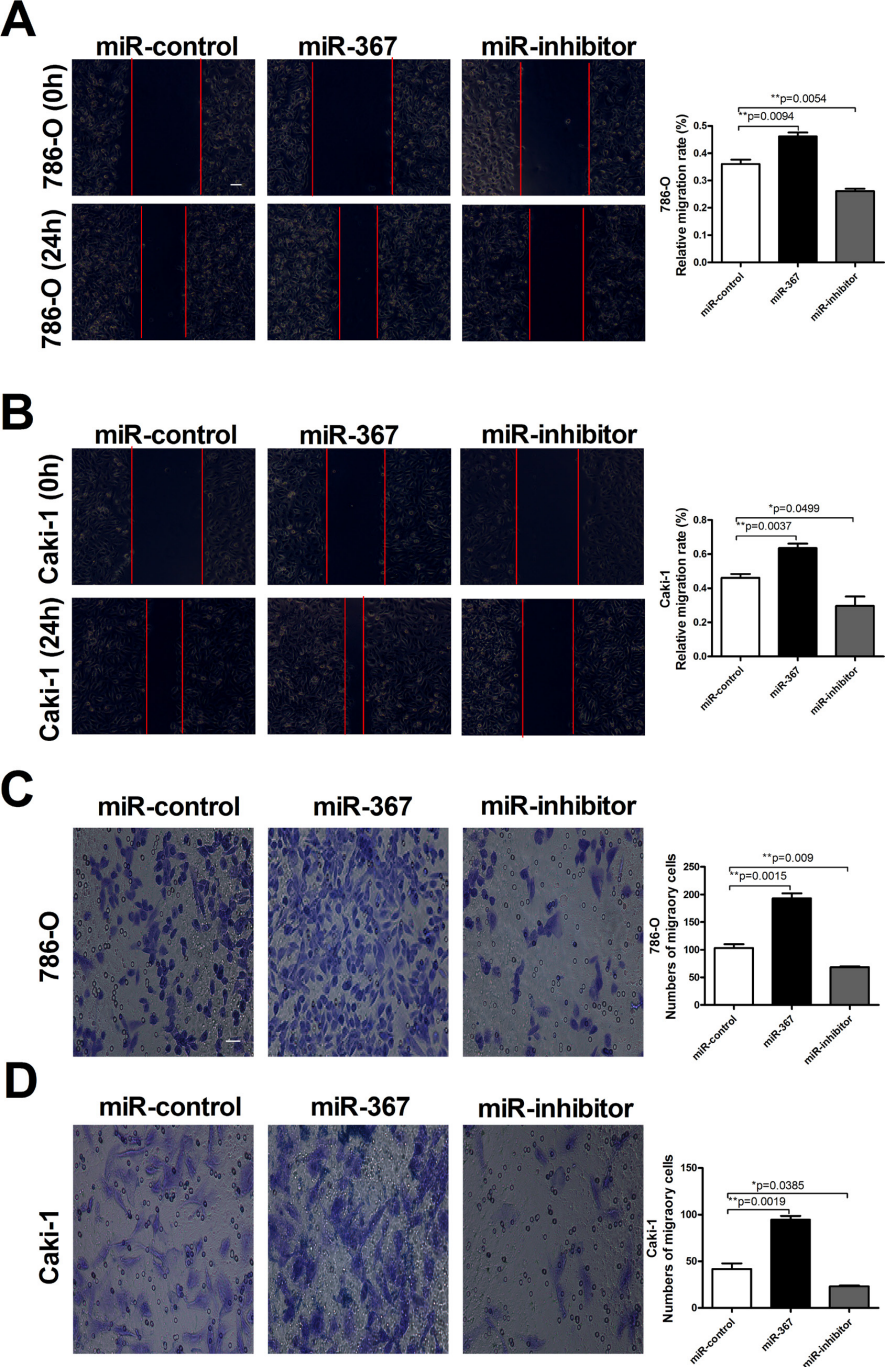
MiR-367 elevation promoted ccRCC cells migration **(A** and **B)** Wound healing assay confirming migration ability of 786-O and Caki-1 cell lines transfected with miR-367 or miR-367 inhibitor. **(C** and **D)** Transwell assay showed the number of migratory cells in 786-O and Caki-1 cell lines in different treated groups. Scale bar indicates 100 μm. Data are expressed as mean ± SEM. **p<0.01 or *p<0.05 *vs*. miR-control.

**Figure 4:**
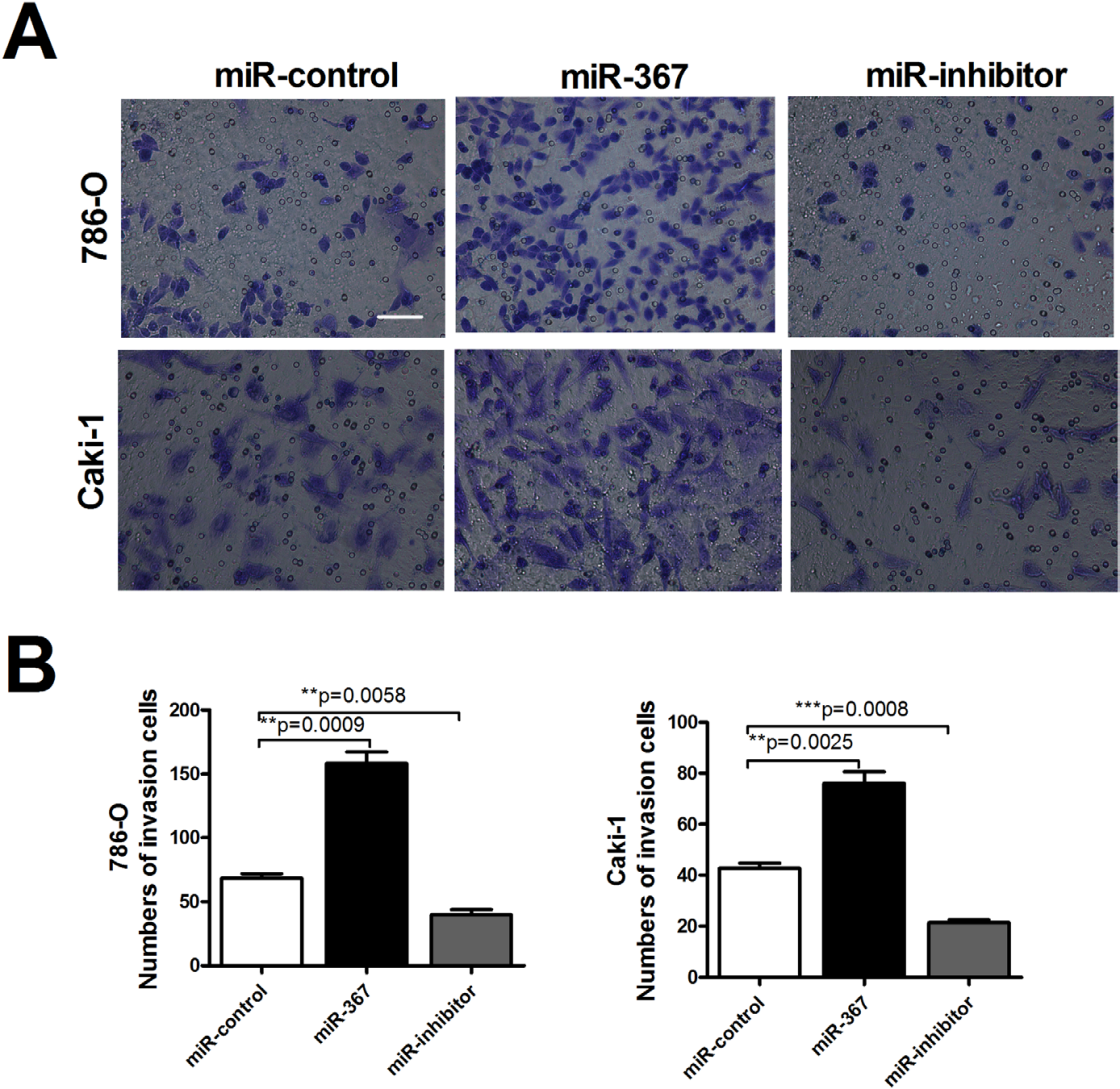
MiR-367 increased the number of invasion cells in ccRCC **(A)** Transwell assays with Matrigel of 786-O and Caki-1 cells transfected with miR-367 or miR-367 inhibitor. Scale bar indicates 100 μm **(B)** Quantitative analysis of the number of invasive cells. Data are expressed as mean ± SEM. **p<0.01 *vs*. miR-control.

Since metastasis of cancer cells is considered as a critical aspect of cancer progression, we detected the effect of miR-367 on migration and invasion capacities of ccRCC cells. The relative migration rates of 786-O and Caki-1 cells were substantially reduced after treatment with miR-367 inhibitor at 24h time point using wound healing assay (Figure 3A and 3B). We further performed transwell assay to assess cell migration. The results also showed that the number of migratory cells were significantly reduced after miR-367 inhibitor administration (Figure 3C and 3D). Transwell assay with Matrigel was employed to evaluate the ability of cell invasion. Elevated level of miR-367 prominently increased cell invasion which could be neutralized by miR-367 inhibitor (Figure 4A and 4B). In addition, to further illuminate the effect of miR-367 on cell migration and invasion, we examined the involvement of miR-367 in Epithelial–mesenchymal transition (EMT) regulation in ccRCC cells. The specific overexpression of miR-367 significantly downregulated the E-cadherin protein level and upregulated the α-SMA protein level, whereas the suppression of miR-367 reversed the results (Supplementary Figure 1A and 1B). These findings confirmed that miR-367 could induce ccRCC cell migration and invasion.

### MTA3 transcription is negatively regulated by miR-367

**Figure 5:**
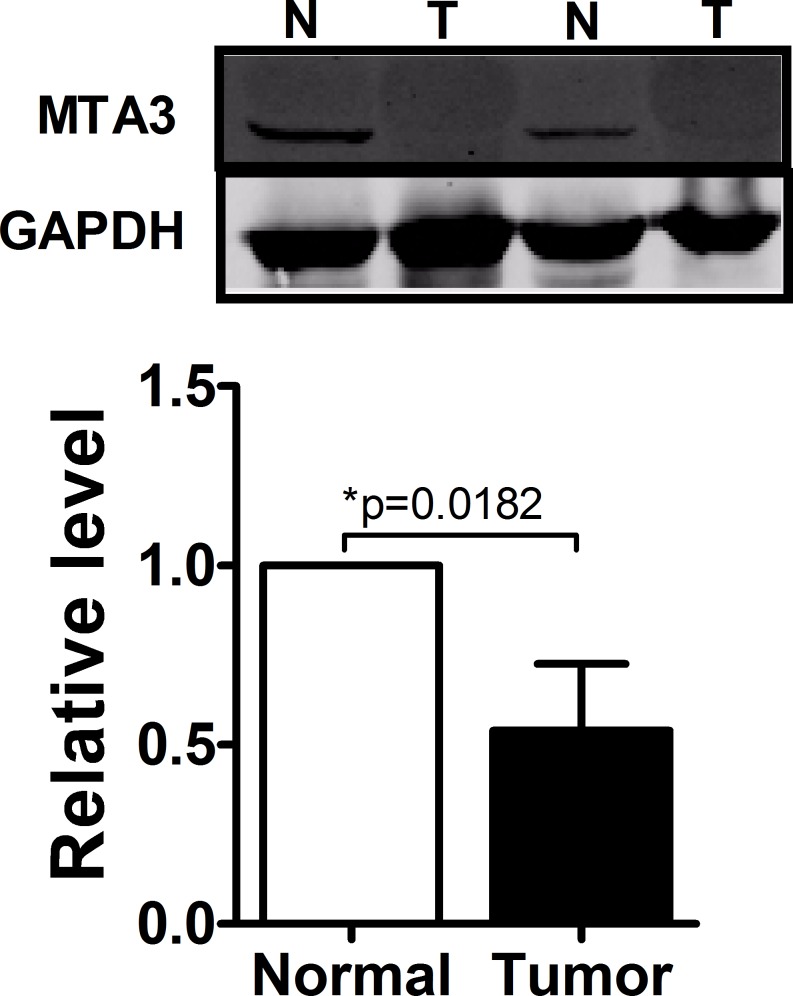
MTA3 was obviously decreased in ccRCC patients Western blot was performed to show that protein level of MTA3 was dominantly decreased in ccRCC patients.

**Figure 6:**
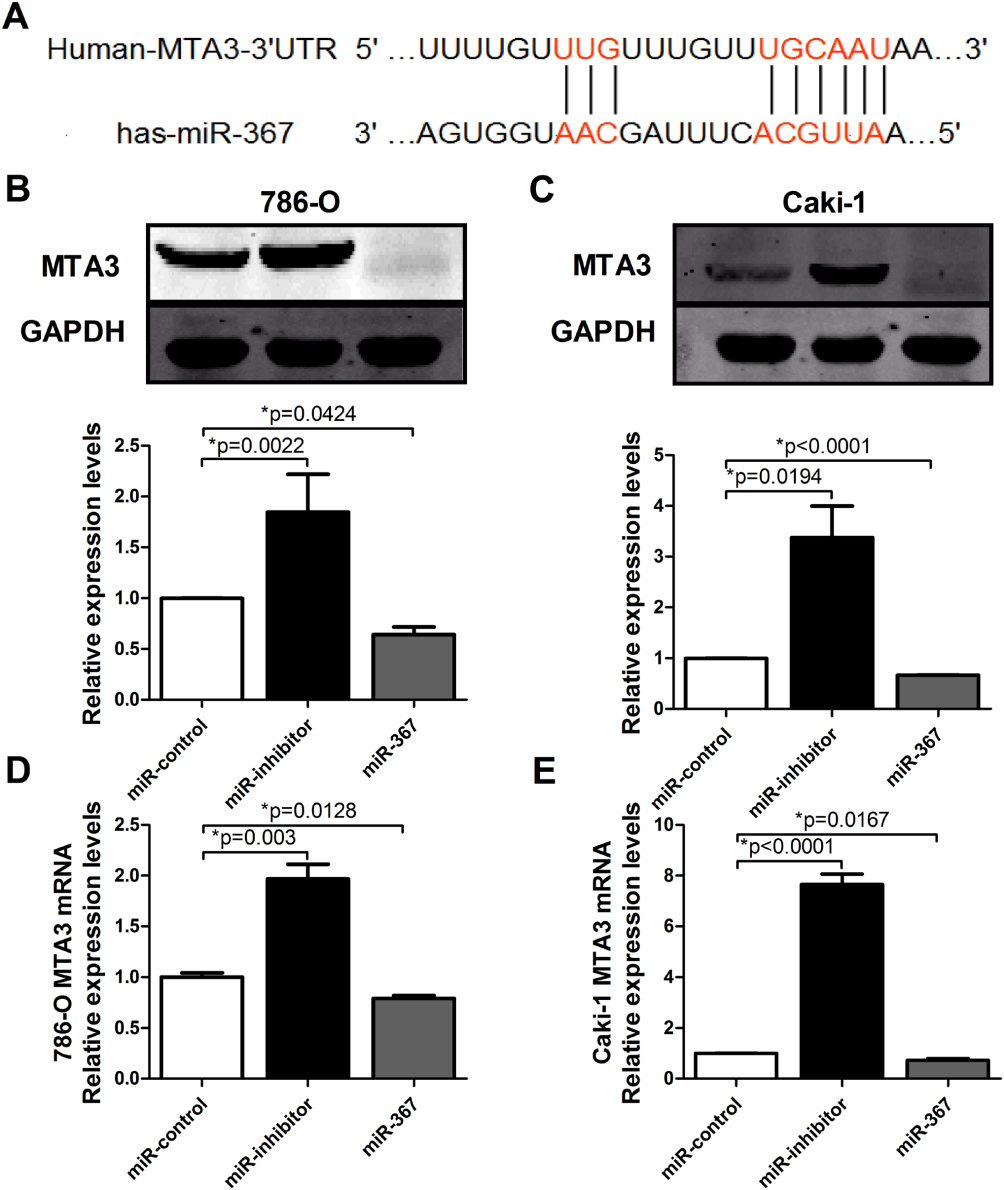
MTA3 is the target of miR-367 **(A)** Bioinformatics prediction was showed. **(B and C)** The protein levels of MTA3 in 786-O and Caki-1 cell lines were analyzed by western blotting. **(D and E)** The mRNA level of MTA3 was measured by qRT-PCR. Overexpression of miR-367 could inhibit the mRNA expression of MTA3. Data are expressed as mean ± SEM. *p<0.05 *vs*. miR-control.

Having established the functional phenotype of miR-367 in ccRCC, we further wanted to identify its target genes to gain insights into the molecular mechanism. Towards this, several bioinformatics predictions were applied, such as TargetScan and miRanDa. All databases predict MTA3 as a potential target for miR-367. Detection of MTA3 expression in ccRCC patients showed that the protein level of MTA3 was substantially decreased in ccRCC patients (Figure 5). The MTA3-encoding mRNA contains a 3′UTR binding site for miR-367 as shown in Figure 6A. Inhibition of miR-367 increased MTA3 protein level both in 786-O and Caki-1 cells, while adverse results were observed when treated with miR-367 mimics (Figure 6B and 6C). As expected, the same results of the mRNA level of MTA3 in 786-O and Caki-1 cells were quantified by qRT-PCR (Figure 6D and 6E). Together, these indicated that MTA3 is a target of miR-367.

### MTA3 could reverse proliferation and metastasis in ccRCC cell

**Figure 7:**
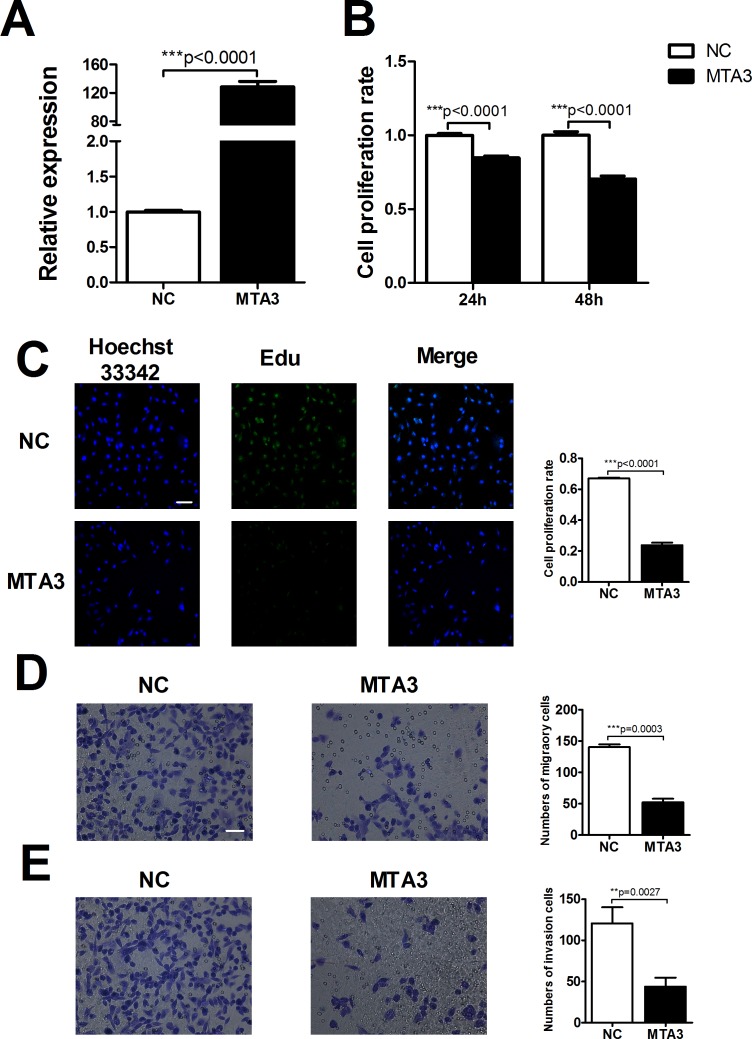
MTA3 is involved in ccRCC proliferation and metastasis **(A)** Transfection efficiency of MTA3 overexpression was assessed by qRT-PCR. **(B)** MTT assay to confirm the ability of MTA3 on ccRCC cell viability. **(C)** After treatment with MTA3 plasmid, the proliferation rate of 786-O cells was determined by EdU kit. **(D** and **E)** Representative micrographs (left) were showed. Relative quantification (right) of cell migration (top) and invasion (bottom) were measured after infected with negative control (NC) or MTA3 in 786-O cells. Scale bar indicates 100 μm. Data are expressed as mean ± SEM. **p<0.01 or *p<0.05 *vs*. NC.

Emerging evidence suggested that MTA3 aberrantly expressed in multiple cancers, including carcinoma of the esophagus, breast, lung, uterus and nasopharynx. This raised the question that MTA3 might play a crucial role in tumors and our interest was directed to explore whether the abnormal of MTA3 was associated with occurrence and development of tumors. Transfection efficiency of MTA3 was first assessed by qRT-PCR suggesting the successful transfection in ccRCC cells (Figure 7A). Western blot was also performed to examine the levels of MTA3 in HK-2, 786-O and caki-1 cell lines. MTA3 was decreased in ccRCC cell lines compared with human renal proximal tubular cells (HK-2 cells) (Supplementary Figure 2). Next, we detected cell viability using MTT and EdU assays, respectively. As shown in Figure 7B and 7C, MTA3 elevation could dramatically decrease cell viability at 24 h and 48h time points. Furthermore, MTA3 elevation was found to significantly reduce migration and invasion of 786-O cells (Figure 7D and 7E). Wound Healing and MTT experiments were carried out to demonstrate inhibition effect of overexpressed-MTA3 on caki-1 cell proliferation and metastasis (Supplementary Figure 3A and 3B). Based on the above data, we concluded that miR-367 mediated proliferation, migration and invasion was, at least in part, attributable to regulate the level of transcriptional and translational MTA3 in ccRCC cells.

## DISCUSSION

In the present study, we found that the expression of miR-367 in tumor tissues was significantly higher than that in matched adjacent normal tissues (Figure 1). In addition, we illustrated that miR-367 played crucial roles in proliferation and metastasis of ccRCC cells. Inhibition of miR-367 could suppress the proliferation, migration and invasion both in 786-O and Caki-1 cells (Figure 2, 3 and 4). As EMT is a key process of tumor invasion, migration, and metastasis [22], the regulation of EMT process by miR-367 further established miR-367 as a pivotal promoter in cell migration (Supplementary Figure 1). Importantly, this is the first study to identify MTA3 as a direct target gene of miR-367. The protein expression of MTA3 was significantly reduced in ccRCC patients. Further studies showed that miR-367 elevation could substantially reduce the protein and mRNA expressions of MTA3 (Figure 5, 6 and 7). Thus, miR-367 may act as a novel type of oncogene by targeting MTA3 in pathophysiologic process of ccRCC.

MiRNAs regulate the expression of target genes at posttranscriptional levels, which induce mRNA degradation or inhibit translation via imperfect hybridization with the 3′-UTRs of target mRNAs [23]. Recent studies suggested that dysregulation of miRNAs was involved in various aspects of cancer progressions [24]. Aberrant expression of miR-367 has been described in multiple types of cancers, such as lung cancer [25], hepatocellular and esophagus cancers [26, 27]. However, little is known about the expression and exact mechanism of miR-367 in ccRCC. To explore the effect of miR-367 on ccRCC, we first analyzed the expression of miR-367 in ccRCC patients. Results showed that miR-367 was significantly upregulated in the tumor tissues. It implied that miR-367 dysregulation was involved in occurrence and development of kidney tumors.

The mechanisms underlying the regulation by miRNAs are often attributed to targeting crucial factors or key signaling pathways [28]. For example, miR-429 regulates cellular function by targeting VEGF [29]. miR-19a promotes ccRCC through downregulation the expression of PTEN, thereby regulating smad4 signaling pathway [30]. miR-30 enhances HIF-2α activity in ccRCC [31]. In our present study, we used two different types of prediction software to predict target genes for miR-367. We found that MTA3 could be a potential downstream target. MTA3 is the latest identified member of metastasis-associated protein family, which also includes MTA1 and MTA2. MTA3 was considered as an estrogen-dependent component of the Mi-2/NuRD transcriptional corepressor in breast epithelial cells [14]. Recently, accumulating evidence showed that MTA3 could regulate cell proliferation and differentiation in various cancers and act as an oncogene in breast cancer and non-small cell lung cancer (NSCLC). Researchers found that MTA3 was dominantly increased in the progress of the above mentioned tumors [32, 33]. In contrast, Dong *et al*. manifested that MTA3 expression was significantly reduced in both tumor tissues and cell lines in gastroesophageal junction (GEJ) adenocarcinoma [34]. Brüning *et al*. found that a significantly lower expression of MTA3 was obtained from 200 patients with endometrioid adenocarcinoma [35]. In this study, serious decrease of MTA3 was observed in ccRCC, indicating that MTA3 played a key role in pathophysiologic process of ccRCC. Further results indicated that overexpression of MTA3 suppressed the proliferation and migration of ccRCC cells and the abnormity of MTA3 was controlled by miRNA-367. Therefore, the potential molecular mechanism could be illustrated by the association between miR-367 and MTA3. However, the effect of miR-367 on ccRCC may be regulated by other genes and pathways as well, and the full mechanisms require our further investigations.

Overall, our study is the first to show induced expression of miR-367 and reduced expression of MTA3 in human ccRCC tissues and cell lines. Targeting MTA3 or inhibiting miR-367 could decrease cell proliferation, migration and invasion. Therefore, miR-367 might represent a novel therapeutic target for treatment of ccRCC patients.

## MATERIALS AND METHODS

### Patients and clinical specimens

Clinical specimens of carcinoma tissues and the corresponding para-carcinoma tissues were obtained from 35 ccRCC patients who had undergone nephrectomy from July 2015 to January 2016 at the Affiliated Tumor Hospital of the Harbin Medical University (Table 1). Written prior informed consent for tissue donation was acquired from each enrolled patient. The protocol was carried out in accordance with the approved guidelines by the Ethics and Scientific Committees of Harbin Medical University.

**Table 1:** Characteristics of ccRCC clinical specimens

	n	(%)
Sex		
Male	23	(65.7)
Female	12	(34.3)
Age		
≤55	14	(40.0)
>55	21	(60.0)
Grade		
G1	8	(22.8)
G2	14	(40)
G3	10	(28.6)
G4	3	(8.6)
Tumor size		
≤5	12	(34.3)
>5	23	(65.7)
T stage		
T1a	4	(11.4)
T1b	21	(60)
T2a	5	(14.3)
T2b	0	(0.0)
T3	3	(8.6)
T4	2	(5.7)

### Cell culture and transfection

Two ccRCC cell lines (Caki-1 and 786-O) were obtained from the Chinese Academy of Sciences (Shanghai, China). Caki-1 cells were cultured in McCoy's 5A medium (Gibco, Grand Island, NY, U.S.) supplemented with 15% fetal bovine serum (FBS; Shanghai Sangon Biological Engineering Technology and Services Co., Ltd., Shanghai, China), and 786-O cells were cultured in RPMI 1640 (Wisent, Saint-Jean-Baptiste, Canada) supplemented with 10% FBS. All the cells were maintained at 37°C in humidified 5% CO_2_ atmosphere. For transfections, cells were starved in serum-free medium for 24 h and transiently transfected with miR-367 mimics, miR-367 inhibitors or negative controls (GenePharma Co., Ltd., Shanghai, China). MTA3 plasmid (RiboBio Co., Ltd., Guangzhou, Guangdong, China) was transferred into cells by using Lipofectamine 2000 reagent (Invitrogen, CA, Carlsbad, USA) according to the manufacturer's protocol. Cells were used for protein/RNA extraction after 24 hours of transfection with the maximum efficiency.

### Cell viability assay

A 3-(4, 5-dimethylthiazol-2-yl)-2, 5-diphenyltetrazolium bromide (MTT) assay was performed to evaluate ccRCC cell viability. Briefly, cells were seeded into 96-well plates followed by miRNA or drug treatments for 24 h. A total of 20 μl of MTT solution was added to each well, and the cells were incubated for 4 h. Thereafter, a total 150 μl of DMSO was added to dissolve the formazan crystals. Absorbance at 490 nm was recorded [20].

### EdU assays

Cells seeded on coverslips in 24-well culture plates were treated as experiment design. The proliferation of Caki-1 and 786-O cells were detected by EdU kit (RiboBio, China) according to the manufacturer's instructions [21]. The stained cells were examined under a confocal laser scanning microscope (FV300, Olympus, Japan). Nuclei that double labeled with EdU and Hoechst 33342 were considered to be positive cells.

### Cell migration and invasion assays

Twenty-four hours after transfection, 1×10^5^ cells in serum-free medium were seeded into the upper chambers of an insert (8-μm pore size; Corning, NY, USA) coated with or without Matrigel (BD Bioscience). The lower chambers were filled with McCoy's 5A/1640 medium containing 10% FBS as a chemoattractant. After 18 h of incubation, the upper side of membrane was cleaned and cells that had invaded through the membrane on the bottom were fixed with 0.1% paraformaldehyde and then stained with 0.1% crystal violet. The migrated cells were observed and counted under a microscope. Cells were seeded in six-well plate with McCoy's 5A/1640 medium to analyze wound healing. After 48 h, the cell monolayer was wounded using a plastic pipette tip and then rinsed with PBS and cultured with serum-free McCoy's 5A/1640 for 24 h. The wound closure was photographed using a microscope(Olympus, Japan).

### Protein isolation and western blot analysis

Western blot analysis was carried out as previously described. The cells were lysed with RIPA buffer containing protease inhibitors phenylmethanesulfonyl fluoride (PMSF). After centrifugation, the supernatant was collected and quantified using the BCA kit (Beyotime, Shanghai, China). Protein samples were then separated by SDS-PAGE and transferred to nitrocellulose membranes which were blocked by 5% non-fat milk for two hours at room temperature. The membranes were rinsed and probed with anti-MTA3 (Abcam, Cambridge, MA, USA) overnight, followed by incubation with florescence-labeled secondary antibody for one hour. GAPDH (Kangcheng Inc., Shanghai, China) was used as an internal control.

### Quantitative reverse-transcription polymerase chain reaction (qRT-PCR)

Total RNA was harvested from tissues and cells using TRIzol reagent (Invitrogen, CA, USA) according to the manufacturer's protocol. The extracted RNA was then reverse transcribed into cDNA using the High-Capacity cDNA Reverse Transcription Kit (Applied Biosystems, Carlsbad, CA, USA; Cat. no. 4368814). The SYBR Green PCR Master Mix Kit (Applied Biosystems) was used to quantify the relative levels of miR-367 and MTA3, with U6 and GAPDH serving as internal controls respectively. The sequences of primers are as follows:

has-MTA3:5′-TCCTCCAGCAACCCATACCT-3′ (forward) and 5′-TCGGTCAAGTCAGCCTCAAC-3′ (reverse);GAPDH:5′-AAGAAGGTGGTGAAGCAGGC-3′(forward) and5′-TCCACCACCCAGTTGCTGTA-3′ (reverse);has-miR-367:5′-CGAGCAATTGCACTTTAG CAAT-3′;andU6:5′-GCTTCGGCAGCACATATA CTAAAAT-3′ (forward)and5′-CGCTTCACGAATTTG CGTGTCAT -3′ (reverse).

### Data analysis

All data in this study were expressed as the mean ± SEM. Statistical comparison between two groups were performed by two-tailed student's t-test and one-way ANOVA followed by Tukey's test were employed to compare three or more means. *p* < 0.05 was considered statistically significant.

## SUPPLEMENTARY MATERIALS FIGURES



## Abbreviations

miR: miRNA, microRNA
MTA3: metastasis-associated protein 3
ccRCC: clear-cell renal cell carcinoma
NSCLC: non-small cell lung cancer
LOXL2: lysyl oxidase like 2
RYR3: ryanodine receptor gene 3
qRT-PCR: quantificational real-time polymerase chain reaction
NuRD: nucleosome remodeling and deacetylase
EMT: epithelial-to-mesenchymal transition
VEGF: vascular endothelial growth factor
HIF2α: hypoxia-inducible factor 2α
PTEN: phosphatase and tensin homolog
GEJ: gastroesophageal junction
