# Supplementary material for: MiR-367 regulates cell proliferation and metastasis by targeting metastasis-associated protein 3 (MTA3) in clear-cell renal cell carcinoma
Source: Oncotarget. 2017 Jun 27;8(38):63084–95. doi: 10.18632/oncotarget.18647 (PMC5609905; doi:10.18632/oncotarget.18647)
Supplement: Supplementary file 1 [file oncotarget-08-63084-s001.pdf]

MiR-367 regulates cell proliferation and metastasis by targeting metastasis-associated protein 3 (MTA3) in clear-cell renal cell carcinoma

SUPPLEMENTARY FIGURES

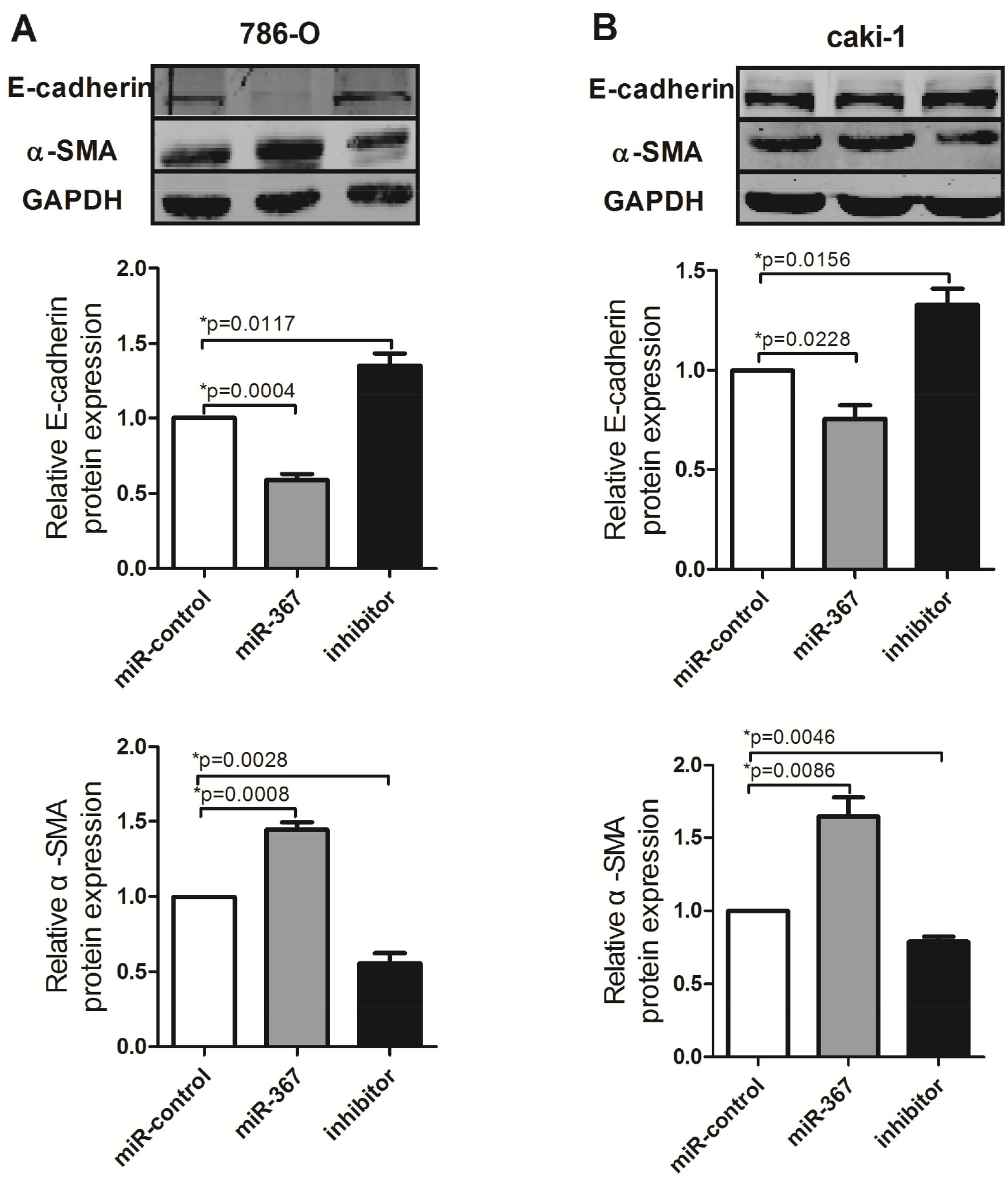

Supplementary Figure 1: EndMT in ccRCC cells is mediated by miR-367. (**A** and **B**) Western-blot was performed to show protein levels of E-cadherin and α-SMA in Caki-1 and 786-O cells. Data are expressed as mean ± SEM; $^{**}p<0.01$ or $^{*}p<0.05$ *vs.* miR-control.

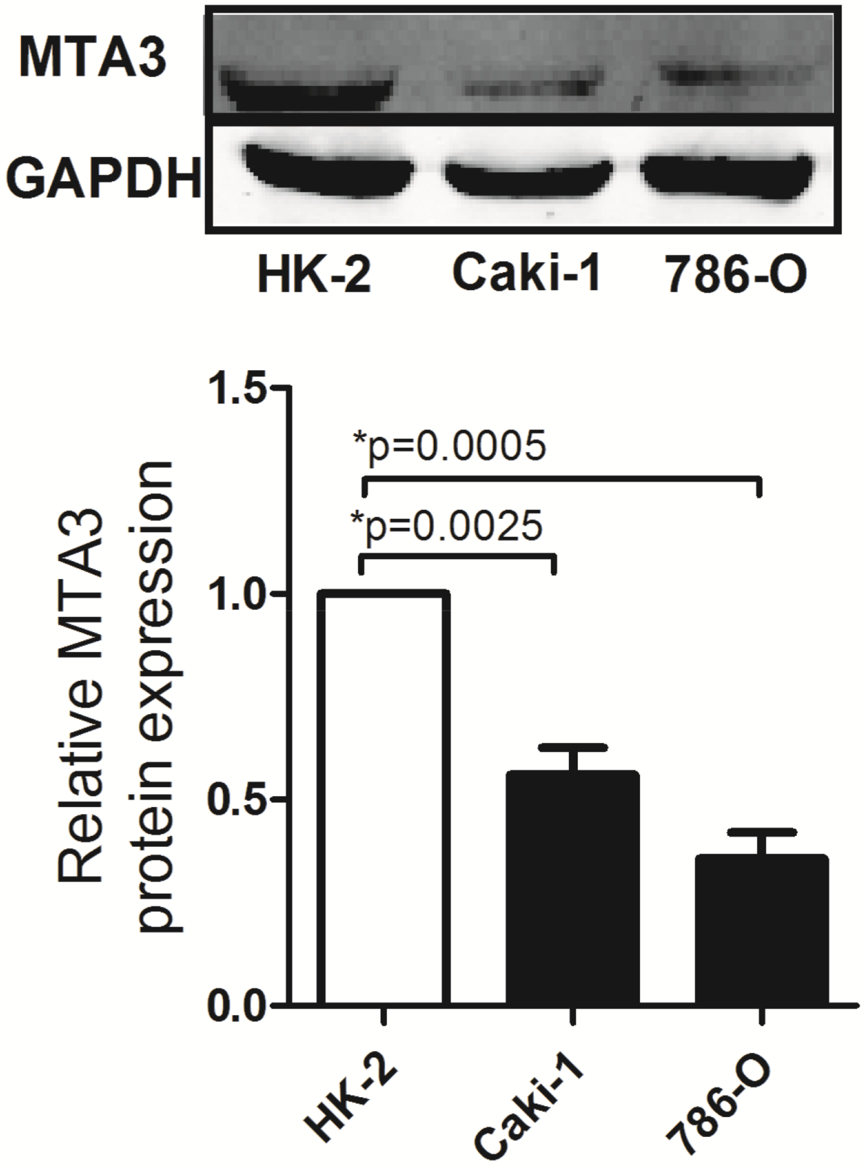

Supplementary Figure 2: Expression of MTA3 in ccRCC cells and human renal proximal tubular cells. MTA3 protein level of ccRCC cells (786-O and Caki-1) and human renal proximal tubular cells (HK-2) were assessed by western blot. Data are expressed as mean ± SEM; ***p*<0.01 *vs.*HK-2.

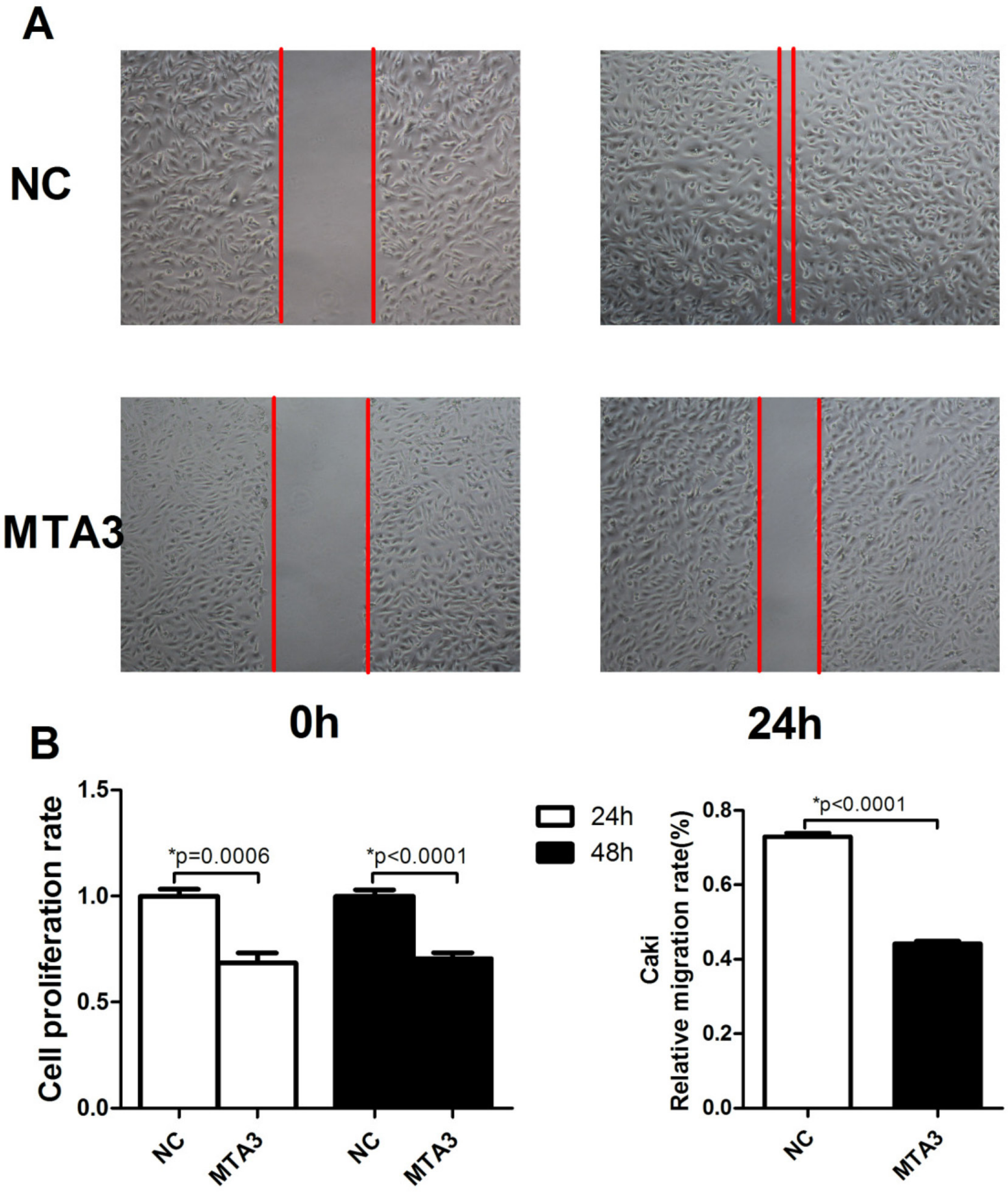

Supplementary Figure 3: MTA3 suppresses caki-1 cell proliferation and metastasis. (A) Wound healing assay to confirm migration ability of Caki-1 cell lines after transfected with MTA3 plasmid. **(B)** Caki-1 cell viability was detected by MTT assay after transfected with MTA3 plasmid for 24 h and 48 h. Data are expressed as mean ± SEM; ***p*<0.01 *vs.* miR-control.
